# Nutritional Modulation of Gene Expression: Might This be of Benefit to Individuals with Crohn’s Disease?

**DOI:** 10.3389/fimmu.2015.00467

**Published:** 2015-09-11

**Authors:** Lynnette R. Ferguson

**Affiliations:** ^1^Discipline of Nutrition and Dietetics, Faculty of Medical and Health Sciences, The University of Auckland, Auckland, New Zealand; ^2^Auckland Cancer Society Research Centre, Faculty of Medical and Health Sciences, The University of Auckland, Auckland, New Zealand

**Keywords:** vitamin D, phytochemicals, probiotics, genetics, genomics, microbiota

## Abstract

The incidence of inflammatory bowel diseases (IBD), including Crohn’s disease (CD), is increasing worldwide, especially in young children and adolescents. Although hospitalized patients are usually provided with enteral or parenteral support, continuing care typically requires a trial-and-error approach to suppressing symptoms and maintaining disease remission. Current nutritional advice does not differ from general population guidelines. International collaborative studies have revealed 163 distinct genetic loci affecting susceptibility to IBD, in some of which host–microbe interactions can be seen to play an important role. The nature of these loci enables a rationale for predicting nutritional requirements that may not be evident through standard therapeutic approaches. Certain recognized nutrients, such as vitamin D and long-chain omega-3 polyunsaturated fatty acids, may be required at higher than anticipated levels. Various phytochemicals, not usually considered in the same class as classic nutrients, could play an important role. Prebiotics and probiotics may also be beneficial. Genomic approaches enable proof of principle of nutrient optimization rather than waiting for disease symptoms to appear and/or progress. We suggest a paradigm shift in diagnostic tools and nutritional therapy for CD, involving a systems biology approach for implementation.

## Introduction

Inflammatory bowel diseases (IBD) cover a spectrum of gastrointestinal disorders, including both Crohn’s disease (CD) and ulcerative colitis (UC), differentiated by their location and behavior. While these diseases were seen as rare in the early twentieth century, they have become increasingly common, causing gut inflammation, ulceration, and other symptoms, in up to 1 in 250 people worldwide. For example, they had not been previously described among Bedouin Arabs in Southern Israel, but numbers are increasing now, almost certainly because of their increasingly urbanized lifestyle (1). A recent commentary described IBD in Asia as the “emergence of a Western disease” (2). While observed previously in Australasia, these diseases are becoming more common in young children and adolescents (3).

There are a number of accepted treatments for CD, aimed not only to reduce the inflammation but also to improve the long-term prognosis (4). The most desirable end point, achieved in only a relatively low proportion of cases at present, is long-term remission. One of the two approaches is used for CD treatment. “Step-up” starts with milder drugs first, while “top-down” gives people stronger drugs earlier in the treatment process. Anti-inflammatory drugs are often the first step in the treatment. These include oral 5-aminosalicylates, such as sulfasalazine and mesalamine, or corticosteroids. However, all of these lead to undesirable side effects. Immune system suppressors also reduce inflammation by targeting the immune system. These drugs include azathioprine (Imuran), mercaptopurine (Purinethol), infliximab (Remicade), adalimumab (Humira), and certolizumab pegol (Cimzia). The latter three drugs are inhibitors of the immune system protein tumor necrosis factor-alpha (TNF-α). It is important to realize, however, that the inflammatory response is a necessary part of response to an infection or injury, and long-term suppression of this may be disadvantageous.

Not only is CD itself debilitating, it also carries with it an increased risk of colorectal cancer (CRC) (5). The nature of this cancer is fundamentally different from the classic disease, which arises from an adenomatous polyp (6). The type of CRC that develops from either form of IBD is almost inevitably lethal. However, like sporadic CRC, it arises through genomic instability.

Genomic instability refers to the accumulation of mutations during the life cycle of cells (7–9). These mutations can involve changes at the level of nucleotide base pairs, chromosome rearrangements, or aneuploidy. Genomic instability can initiate cancer, augment cancer progression, and influence the overall prognosis of the affected patient. It arises from many different pathways, including telomere damage, centrosome amplification, epigenetic modifications, and DNA damage from endogenous and exogenous sources. Protection against genomic instability, or at least reduction of the probability, is an important function of certain nutrients and phytochemicals (7).

Although nutrition is an important consideration in hospitalized patients, non-hospitalized patients are not currently being given nutritional guidelines outside those for the general population. We explore the possibility that thinking outside the current square in terms of nutrition might have significant beneficial effects beyond protection from malnutrition. That is, we suggest that nutrition could move from being an adjunct to therapy, to the point where it becomes primary therapy in its own right, integrated with currently accepted approaches (10). There is reason to suggest that this could have significant benefits in terms of delaying IBD progression and reducing the possibility of CRC formation. Such information would ideally be tailored according to individual patient genetics, and validated using systems biology approaches, as detailed in the following sections.

## Diet in Non-Hospitalized CD Patients

Either prior to hospitalization or following discharge from hospital, dietary selection is determined by something of a random process (11). While the diet may be based on previous recollections of foods which affected symptoms, either in a positive or negative sense, it is often guided by advice from others with the disease. It has been clear for some time that certain foods may have adverse effects in most subjects, exacerbating disease symptoms by increasing inflammation and/or producing flatulence. In some groups, there has been endorsement of a “low FODMAP” diet, as this appears to reduce some of the symptoms in CD and other functional gastrointestinal disorders, especially irritable bowel syndrome (12, 13). FODMAP refers to the combination of saccharides and polyols and is an acronym of “Fermentable Oligo-, Di-, mono and polyols.” Against the recommendation for a low-FODMAP diet, there is a concern that such a structured dietary regime will have adverse effects in reducing dietary diversity. Of particular concern is the recognition that this same group of excluded nutrients plays a major role in modulating the composition of the gut microbiome (14).

It has been recognized that there is a considerable danger of malnutrition exacerbating the already difficult symptoms of CD. Attention has been drawn to the possible need for higher than normal levels of vitamins such as vitamin D or lipids such as long-chain omega-3 polyunsaturated fatty acids (*n*-3 PUFAs) (15, 16). What has not always been recognized is the role of dietary components that are not currently included in essential lists. For example, prebiotics and probiotics may be important in regulating the gut microbiota, and their importance may be missed in some assessments (17–19). Phytochemicals are sometimes called “non-nutrients.” Although these are not included in lists of essential nutrients, many of them play key roles in maintaining genomic stability and in modulating the composition of the gut microbiome (7, 20, 21). These could be key factors in helping to prevent the development of CRC in IBD.

Irrespective of nutrient composition, it has repeatedly been observed that foods considered to be beneficial for some individuals may actually cause adverse effects in others (11, 22). Individuals with the diseases differ in genetic, epigenetic, and phenotypic characteristics, so it is not surprising that a single approach is unlikely to be beneficial to all. However, studies showing the importance of genotype in the phenotypic characteristics of the diseases encourage the possibility of tailoring diets based on genetic and genomic information (23).

## Genetic Basis of CD

Crohn’s disease was one of the subjects of early reports on genome-wide association studies (GWAS). At that time, it appeared that there could be up to eight loci affecting disease susceptibility (24). Since that time, large international groupings have performed a meta-analysis of CD and UC GWAS scans, as part of activity of the International IBD Genetics Consortium (IIBDGC) (25). This was followed by extensive validation of significant findings, from data on a combined total of more than 75,000 cases and controls. These efforts initially revealed 163 distinct genetic loci affecting susceptibility to IBD overall (26), but this number has more recently been expanded to 201 (27). A number of these genes are involved in primary immune deficiencies, characterized by a dysfunctional immune system and increased susceptibility to serious infections. In particular, there is a considerable overlap between IBD loci and the immune-mediated disorders, ankylosing spondylitis and psoriasis (27). This study found considerable overlap between susceptibility loci for IBD and mycobacterial infection, which relationships were emphasized by further analyses using coexpression network analysis. Many of the identified loci contain genes involved in microbial handling, and host–microbe interactions clearly play an important role in disease susceptibility (26).

In further analyses of these datasets, the IIBDGC detailed fine-mapping project, clarified the nature of these genes and their interactions, and suggested that phenotypically there should be three rather than just two main classes of IBD and that CD should be categorized into two distinct classes depending upon its location (27). Each of these classes of disease has characteristic genetic and phenotypic characteristics. The detailed nature of the genes and networks involved in disease susceptibility enables a rationale for predicting nutritional requirements that may not be evident through standard therapeutic approaches.

## Possible Deviations from Conventional Nutrient Requirements in CD

**Figure 1:**
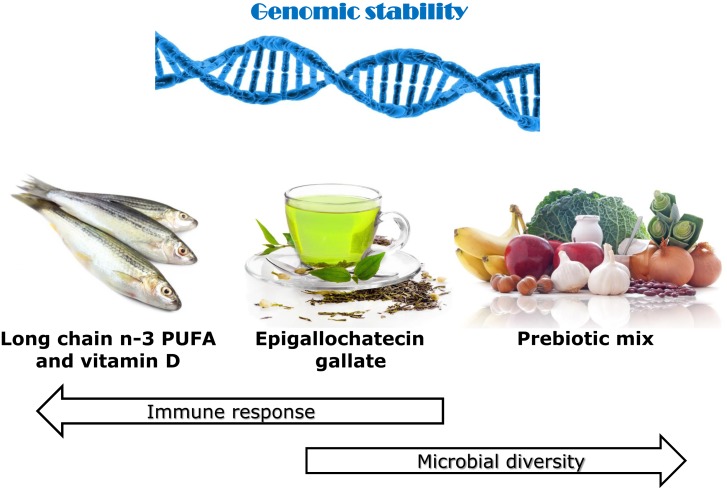
**Effects of various identified dietary components on immune response, microbial diversity, and genomic stability, with use justified by genetic testing**. The use of the immunochip supplemented with fine mapping enables very detailed understanding of the nature of the genes involved in CD susceptibility of an individual. A number of the affected genes play key roles in inflammatory response. If the majority of the affected genes favor inflammation, then a primary recommendation may be to enhance the intake of long-chain *n*-3 PUFA, especially EPA and DHA. Both of these play distinctive and major controlling roles in inflammatory response (16). If genes affecting microbial response and signaling pathways predominate, then probiotics may be an especially important dietary component (35). In addition, various prebiotics such as the food combination illustrated here act to stimulate the growth of beneficial gut microbes (14, 37). Both vitamin D and various phytochemicals, including the active component in green tea, EGCG, are recognized as having roles in each of these processes and are likely to be beneficial to CD patients at higher than standard dietary recommendations (15, 38–40). Maintenance of genomic stability will play an important role in slowing progression of CD or the development of CRC (7, 21). Thus, diets containing a good balance of such components, possibly tailored according to genotype, may act to complement or slow the need for recognized therapeutics in CD. Images from Can stock photos.

There is a considerable scientific input into the setting of recommended daily nutrient intakes. While these may be good for the general population, they may often not reflect the actual requirements for CD patients. Figure 1 illustrates the identification of specific dietary components or items that may be important for different groups of CD patients, according to their genotype and the nature of the gut microbiome. Genetic testing can indicate the primary single-nucleotide polymorphisms (SNPs) of importance at an individual level. If, for example, some of the interleukin (IL) genes are affected, these are primarily involved in inflammation (28, 29), although they may have a downstream effect on the microbiota. Thus, supplementation with long-chain *n*-3 PUFA or fish oils may be a nutritional approach to prioritize (30–32). Similarly, identification of NOD2 or ATG16L1 gene variants indicate that ability to respond appropriately to colonic bacteria is compromised (33, 34), and prebiotics and/or probiotics may provide an important starting point (14, 35–37). For all these individuals, especially those carrying a number of variant SNPs, it becomes important to protect against genomic instability since this will reflect a propensity for cancer development. In this respect, certain nutrients, especially vitamin D and phytochemicals, may play an important role (7).

While it is desirable to have studies in CD patients, in practice much of this work comes from mouse models, developed to reflect our understanding of the genetics of CD (41–44). Two of these models will be described here.

The IL-10 gene-deficient (*IL-10^−/−^*) mouse model lacks a functional version of the anti-inflammatory cytokine, IL-10. *IL-10^−/−^* mice, inoculated with normal intestinal bacteria, have been used to investigate the role of various dietary components in intestinal inflammation, including mechanistic studies that consider transcriptomic, metabolomic, and proteomic effects (41, 45, 46). The multidrug-resistant (*mdr1a^−/−^*) mouse model carries a deletion mutation enabling disease-causing microorganisms (bacteria, viruses, fungi, or parasites) to resist a range of important drugs (antibiotics, antifungal, antiviral, and antiparasitic drugs) targeted at eradicating the organism (47). *Mdr1a* knockout mice are susceptible to developing a severe spontaneous intestinal inflammation in pathogen-free animal facilities.

### Micronutrients

Fenech has reviewed the role of various micronutrients in slowing the progress of genomic instability, a key component in the progression of digestive diseases and the initiation of cancer (8, 48, 49). He points to the importance of individualizing dietary components according to genotype and shows increased dietary intake of vitamin E, calcium, folate, retinol, and nicotinic acid being associated with less DNA damage and a need to define the optimal amount being especially important for riboflavin, pantothenic acid, and biotin. These three have been distinguished because of increased DNA damage being especially evident at higher doses. Fenech has described high-throughput nutrient arrays that enable defining, on an individual basis, the optimal combination of nutrients for DNA damage prevention, maintenance of telomere integrity (important in cancer risk), and cancer growth control (48).

We have more generally reviewed vitamin and mineral requirements to maintain genomic stability, especially in the context of the micronutrient genomics project (50). It is noteworthy that certain of these nutrients may be required in higher than usual amounts in CD, since they are utilized in the control of immune response and inflammation. Our own studies have particularly emphasized the importance of getting not only the correct form of selenium but also the appropriate level according to genotype (51, 52).

Vitamin D is an important vitamin that appears to be required at higher than anticipated levels in CD patients (15). This may partly be caused by genetic requirements, and it is of interest that a number of SNPs associated with vitamin D uptake and distribution actually appear on the immunochip, used in the important study of IBD risk genes by Jostins and coworkers (26). Again, there is a specific link with inflammatory processes and with control of microbiota in the identified genes. Higher plasma vitamin D levels have been associated with a reduced risk of *Clostridium difficile* (53), whereas reduced levels of circulating vitamin D enhance the risk of cancer and other inflammatory diseases (54, 55). We suggest that such an observation may add fuel to an argument that higher than current recommended daily intakes of vitamin D may be particularly appropriate to CD patients.

### Dietary lipids

Many naturally occurring agents directly bind with and activate peroxisome proliferator-activated receptor gamma (PPAR-γ or PPAR gamma), a type II nuclear receptor that in humans is encoded by the PPAR-γ gene. Agents binding this include various PUFAs including arachidonic acid and arachidonic acid metabolites. PPAR-g regulates fatty acid storage and glucose metabolism. The genes activated by PPAR-g stimulate lipid uptake by adipocytes and play an important role in regulating inflammation and cancer cell growth (46).

Peyrin-Biroulet and coworkers (56) demonstrated antimicrobial functions of the PPAR-γ gene products in maintaining epithelial expression of a type of colonic beta-defensin (mDefB10 in mice, DEFB1 in humans). In mutant mice carrying this mutation, these authors showed defective killing of a number of bacteria including *Candida albicans*, *Bacteroides fragilis*, *Enterococcus faecalis*, and *Escherichia coli*. It appears that colonic involvement in CD is linked to reduced expression of DEFB1, independent of inflammation. Thus, it has been suggested that PPAR-g-targeting by either drugs or nutrients could prevent colonic inflammation by restoring antimicrobial immunity in CD.

There have been variable results across various studies in relation to the association of common PPAR-g variants (C161T and Pro12Ala) with IBD. While Hume and coworkers reported no association in an Australian cohort (57), Shrestha et al. found suggestive relationships in Chinese but not Dutch patients (58). However, the Pro12Ala variants appeared to be protective against the development of CD in European Caucasians (57). Such studies have led support to the suggestion that there are significant ethnic differences in the phenotypic expression of this variant. There is reason to believe that diet, including lipids, may play a key role in this effect.

Conjugated linoleic acid has been shown to modulate immune responses in patients with mild to moderately active CD (59), and there is some support for this being through modulation of PPAR-g. In their Caucasian CD cohort, Ferreira and coworkers (60) found that a high intake of total, saturated, and monounsaturated fats and a higher ratio of *n*-6/*n*-3 PUFAs were associated with a more active phenotype. They studied the effects of four genetic polymorphisms, including the two PPAR-g SNPs 161C/T and Pro12Ala SNPs, in a case–control population. Although they reported no significant effects of these SNPs on disease susceptibility *per se*, they found that the presence of either of these SNPs led to a more detrimental effect of a high intake of total and trans fats.

The effects of omega-3 polyunsaturated fatty acids (*n*-3 PUFAs) are also modified by genotype, especially the nitric oxide synthase (NOS3) gene (61). These authors reported associations of plasma fatty acid composition and NOS3 SNP genotypes (rs11771443, rs1800783, rs1800779, rs1799983, rs3918227, and rs743507) in 450 individuals with the MetS from the LIPGENE dietary intervention cohort. They found that several markers of inflammation were significantly different between the genotype groups. There was a significant gene–nutrient interaction between the NOS3 rs1799983 SNP and plasma *n*-3 PUFA status on plasma triacylglycerol (TAG) concentrations. Minor allele carriers (AC + AA) were considerably more responsive to changes in plasma *n*-3 PUFA than major allele homozygotes. Such individuals are likely to benefit even more from long-chain *n*-3 PUFA consumption than the general population in order to reduce inflammation in CD (16).

### Phytochemicals

Polyphenols are secondary metabolites of higher plants and one of the largest groups of natural products. Although not usually considered in the same class as essential nutrients, various polyphenols could play an important role in CD susceptibility and progression. A range of polyphenols have been shown to modulate inflammation, especially in genetically deficient mouse models. For example, a green tea extract enriched in polyphenols was able to modulate colonic inflammation in mice lacking the multidrug resistance gene (*Mdr1a^−/−^*). Mice were fed control or green tea-enriched diets for 21–24 weeks of age, after which a colonic histological injury score was obtained, colonic gene expression analyzed using microarrays, and colon protein expression also measured (20). The authors reported reduced abundance of transcripts and proteins associated with immune and inflammatory response, which suggested that its anti-inflammatory activity is mediated by multiple molecular pathways.

Cruciferous vegetables, such as cabbage and broccoli, contain two types of sulfur-containing phytochemicals – glucosinolates and *S*-methyl cysteine sulfoxide. While these chemicals have a range of effects generally considered to be beneficial in the population at large, this group of food plants also polarizes individuals with CD. Laing and coworkers associated SNPs and the beneficial or adverse effects of the 10 most commonly eaten foods in this group (22). One of the SNPs that showed exceptionally beneficial properties to individuals consuming cruciferous vegetables was in the defensin (DEFA6) gene. Conversely, one SNP strongly associated with adverse effects was in the major histocompatibility complex, which characterizes one important group of CD patients (27).

The main polyphenol in green tea is (−)-epigallocatechin gallate (EGCG). Unno and coworkers showed this compound at levels of 0.6% to have strong effects on gut microbiota and biomarkers of colonic fermentation in rats (38). They found a significant reduction of *Clostridium* spp and an increased gut occupation by *Bacteroides*. Smaller changes were seen for *Bifidobacterium* and *Prevotella*. ECGC also has effects on genomic stability, suggesting that it could be protective against cancer development in IBD (7, 21).

Ellagic acid and ellagitannins are a class of hydrolyzable tannins found in some fruits and nuts. At least in *in vitro* studies, there is good evidence that these may have the potential to reduce inflammation in genetically modified cells (62). Boysenberries, a hybrid *Rubus* berry, are among the best food sources of ellagitannins, although chestnuts and pomegranates also have high concentrations. Nasef and coworkers found effects modulated through toll-like receptors 2 and 4 of extracts from blackcurrants and feijoa, which may have been associated with elligitannins (63). The significance of these results awaits confirmation by *in vivo* testing.

Resveratrol is another polyphenol that has been shown to protect against genomic instability. It acts as an antioxidant protecting against free radical-induced DNA damage and is likely to play a protective role in gut inflammation and progression to CRC (64).

### Probiotics and prebiotics

The diversity of microbiota appears as a key characteristic that may predict response to therapy in CD (65). In addition, it has been suggested that a discriminant score of intestinal microbiota may provide an index of disease activity in CD (66). While fecal transplants have received some recognition as therapy for CD generally, these are not sustainable over a long period (67). Additionally, a number of concerns have been raised about their true efficacy (68). Regulating the gut microbiota through diet may provide a more sustainable solution. There is no question but that diet plays a major role in modulating the colonic microbiota (69). Certain nutrients, especially vitamin D, may play an important role in this respect (70).

Probiotics have been defined by FAO/WHO as “live microorganisms that confer a health benefit to the host when administered in adequate amounts.” Especially in combination with prebiotics (plant substrates that enable modulation of colonic microbiota), these may be especially beneficial to CD patients (35, 71). Probiotic bacteria have been shown to produce conjugated linoleic acid in the gut, which in turn plays a role in suppressing disease symptoms through targeting PPAR-γ gamma (42). Prebiotics are digested and fermented to form short-chain fatty acids, which themselves enhance the growth of certain important colonic bacteria (17).

Additionally, an increasing number of plant polyphenols are being identified to have effects on colonic microbiota identification and regulation (64). Innovative prebiotic/probiotic foods are also being developed and may have promise in a clinical context (71).

## Methylation, miRNAs, and CD

Not only genetics *per se* but also certain epigenetic events are associated with susceptibility to CD, disease progression, and CRC development.

DNA methylation has been considered for its role in the development of CD. Whole-blood DNA methylation profiles were compared in treatment-naive children with CD and healthy controls, as measured using the Illumina 450 K platform (72). Sixty-five differentially methylated CpG sites were identified as reaching epigenome-wide significance. The most significantly differentially methylated region in patients with CD involves the transcription start site for microRNA (miR)-21.

microRNAs (miRNAs) are recognized as playing an essential role in the development and control of the innate and adaptive immune system. These are small non-coding RNA molecules that lead to post-translational gene silencing and control gene expression in diverse biological processes, including inflammation. miRNA genes occur within intronic sequences of protein-coding genes, within intronic or exonic regions of non-coding RNAs, are intergenic (72). As well as miR-21, various other miRNAs including miR-192, miR-122, miR-29, and miR-146a may be implicated in CD. Krissansen and coworkers found that increased circulating levels of miR-595 and miR-1246 related to a highly aggressive form of the disease (73). Because miRNAs play a major role in regulating gene expression, they are being looked at as therapeutic targets and also as biomarkers for aggressive disease development.

## Effects of Selected Nutrient Classes on Gene Expression in CD

There are no published reports of individual nutrients affecting gene expression in CD from human studies because the definitive studies would be unethical to perform. However, there are a number of studies in animal models or tissue culture systems, which provide useful information.

### Micronutrients

The active binding sites of 1,25-dihydroxyvitamin D3 (1,25D) are likely to regulate gene expression at the cellular level (74). This active form of vitamin D has been shown to interact with the epigenome through effects on DNA methylation, histone acetylation and miRNA, as well influencing pre-mRNA splicing. Genomic profiling has identified a set of 1,25D regulated genes that are especially relevant to cancer prevention. For example, Ma and coworkers compared 1,25D-regulated miRNA expression profiles in a human cancer cell line in comparison with a highly tumorigenic and metastatic variant, using miRNA qPCR panels (75). The two lines showed distinctly different miRNA expression profiles that the authors suggest are likely to influence the behavior of different tumor cells and are relevant to both the susceptibility to and subsequent behavior of cancer.

Human studies on vitamin D3 supplementation have enabled dissection of high from low responders in a pre-diabetic study population (76). Only around 60% of these subjects responded to supplementation. While VDR receptor gene polymorphisms played a role in this, other clinical parameters such as the level of parathyroid hormone were also important. The authors suggested that vitamin D3-induced changes in human peripheral blood mononuclear cells can be described by transcriptomics. Added to information from other serum biomarkers, these allow the identification of those subjects who will (or will not) respond to vitamin D supplementation.

### Long-chain omega-3 PUFA

These compounds have sometimes been described as master regulators of gene expression (16). Serhan and coworkers have discussed the function of long-chain omega-3 PUFA as novel pro-resolving mediators in the resolution of acute inflammation. They have discovered a new genus of pro-resolving lipid mediators that is temporally produced by resolving exudates that enable a return to homeostasis. These not only have anti-inflammatory actions but also enhance microbial clearance. Such properties make the two long-chain omega-3 PUFAs, EPA and DHA, essential nutrients not only for protection against CD in the first place but also for reducing inflammation and thereby reducing disease progression.

Knoch and coworkers compared the effects of EPA supplementation with that of oleic acid (OA) as control in the *IL-10^−/−^* mouse model (77). They found that EPA reversed the decrease in colon fatty acid beta-oxidation gene expression observed in OA-fed mice. The mice fed with the OA diet showed a decrease in the expression of antioxidant enzyme genes, as well as those involved in detoxification, when compared with wild-type (C57Bl) mice on the same diet. In contrast, EPA up-regulated the expression of these same enzymes. The authors suggested that these results imply that EPA might have a potential anti-inflammatory effect on colon tissue. In support of this hypothesis was the observation that EPA also activated expression of the PPAR-α gene, which regulates the expression of proinflammatory and immunomodulatory genes.

### Phytochemicals

Cytochrome P450s (CYPs) play an essential role in the metabolism of endogenous and exogenous molecules (78). They facilitate the biosynthesis of a number of essential molecules, including fatty acids, lipid-soluble vitamins, and steroid hormones. They also metabolize most pharmaceuticals as well as carcinogenic compounds. CYP gene expression is regulated by a number of polyphenols, such as EGCG. This molecule not only has antioxidant properties but can be pro-oxidant at very high levels, although generally beneficial in modulating the risk and progression of inflammatory diseases and cancer (79). The review by Korobkova extensively summarizes the effects of a range of polyphenols on CYP metabolism, with flow-on implications for gene expression (79). Aylissi and coworkers have also summarized the epigenetic effects of natural polyphenols, which have implications for the modulation of gene expression (80).

An extract of green tea, enriched in polyphenols (GrTP), was tested for effects on colonic inflammation in the MDRa*^−/−^* mouse model utilizing a transcriptomics approach (20). The histological injury score was significantly lower in the GrTP-fed mice than in the control group. Colon mRNA transcripts were assessed using microarrays. They revealed a reduced abundance of transcripts associated with immune and inflammatory response and an abundance of those associated with xenobiotic metabolism pathways, suggesting an anti-inflammatory effect mediated by diverse mechanisms. PPAR-a and signal activator of transcription 1 (STAT1) appear as two key regulatory molecules in these events.

Curcumin has been studied for effects on gene expression, using the N2A cell line (81). Treatment with this polyphenol leads to suppression of NF-kappaB and its downstream proinflammatory targets including COX-2 and iNOS. Resveratrol has also been shown to alter gene expression patterns in another cancer cell line (82). Microarray analysis showed effects on apoptosis-related genes. The green tea polyphenol, EGCG, has been used as an exemplar of a polyphenol showing effects on gene expression revealing other than antioxidant properties (39). In particular, EGCG regulates signal transduction pathways, transcription factors, DNA methylation, mitochondrial function, and autophagy, several of which effects are relevant to CD patients. ECGC has also been shown to attenuate the activation of STAT3, which again is an important pathway in CD (83). In an overview on the effects of polyphenols on gene expression, Joven and coworkers suggest that these provide an excellent illustration as to how we may be able to eat our way out of chronic diseases (84).

Dietary curcumin and rutin were compared for effects on colonic inflammation and gene expression in the MDR1*^−/−^* mouse model (44). Curcumin but not rutin significantly reduced the histological evidence of inflammation in this mouse model. Microarray and pathway analysis implied that the mechanism was likely through up-regulation of xenobiotic metabolizing enzyme expression and a down-regulation of proinflammatory pathways.

### Probiotics and prebiotics

Plaza-Diaz and coworkers found evidence that a range of probiotics modulate the expression of immunity and inflammatory genes in the gut, which appear especially relevant to CD. They performed a systematic review to conclude that strains of *Bifidobacterium*, *Lactobacillus*, *Escherichia coli*, *Propionibacterium*, *Bacillus*, and *Saccharomyces* influence the gene expression of mucins, Toll-like receptors, caspases, NF-κB, and ILs, leading to an anti-inflammatory response in cultured enterocytes. Similar results were found in animal models ranging from fish to mice, rats, and piglets (85).

Prebiotics can also modulate host gene expression, with potentially beneficial flow-on effects relevant to CD (86). Sauer and coworkers studied the effects of products formed from an inulin derivative incubated with human gut flora (87). They considered the expression of 96 genes related to biotransformation using cDNA microarrays. The pattern of gene expression suggested various effects likely to protect the cells from carcinogenic compounds. Zenholm et al. used a Caco-2 gastrointestinal cancer cell model to study the effects of an oligosaccharide and a fructo-oligosaccharide in reducing the expression of IL-12p45, IL-8, and TNF-α (88).

## Human Studies on the Benefits of This Combination of Nutrients on Gene Expression

Marlow and coworkers reported on gene expression effects of a specific diet containing examples of the nutrient classes under discussion (89). They developed a Mediterranean-inspired diet whose ingredients were delivered to participants each week over a 6-week intervention period along with recipes and tips for maintaining a healthy diet, and participants were also contacted for encouragement on a regular basis. The diet did not contain the amounts of whole grains and diary products common in the Mediterranean but had a seasonal range of fruits and vegetables (various vitamins and polyphenols), two meals of oily fish per week (omega-3 and vitamin D), green tea (EGCG), and probiotic capsules. Marlow and coworkers considered the effects on symptoms, biomarkers of inflammation, and the gut microbiota as well as classic measures of inflammation such as C-reactive protein (CRP), and they also used a transcriptomic end point. They found that adherence to the diet for 6 weeks reduced the established biomarkers of inflammation. However, transcriptomic analysis provided an important biomarker, showing that the expression of certain genes important in the etiology of CD was beneficially modulated. The diet also resulted in a trend of normalizing the microbiota.

## Systems Biology Approaches to Validate the Efficacy of Dietary Approaches to Disease Control

**Figure 2:**
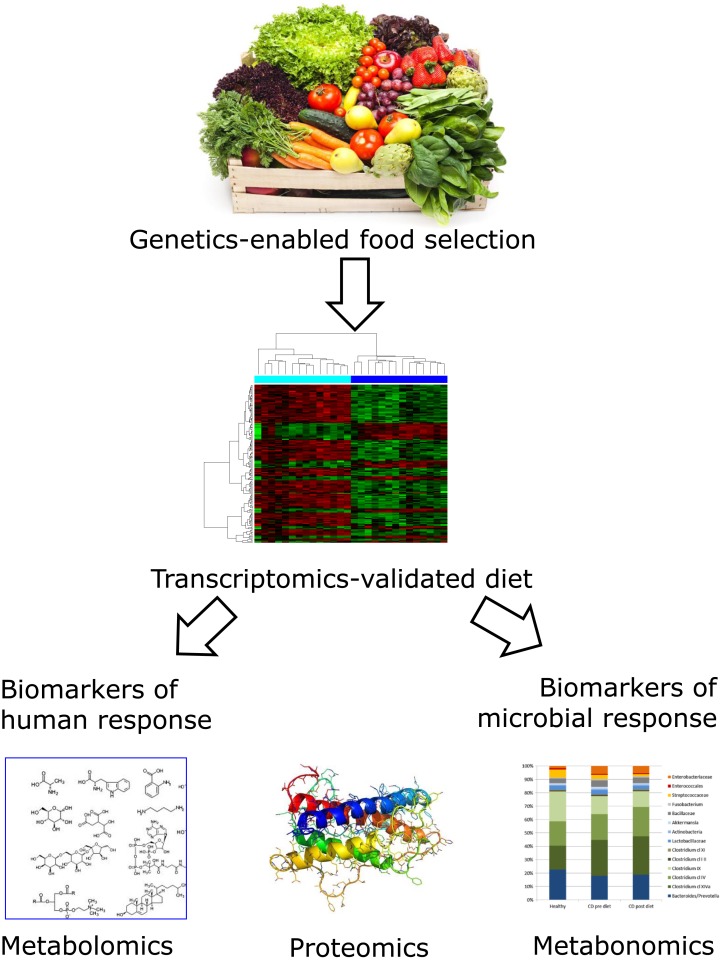
**Flowchart showing how genetic and genomic technologies may inform optimal nutritional modulation of CD**. Genetic characterization of human DNA and the nature of the associated gut microbiome enable selection of an individualized diet for CD patients. While long-chain omega-3 PUFA containing fish or dietary supplements may be appropriate for most individuals, the exact nature of the most desirable fruits, vegetables, and supplements will vary. Although we are now in a position to make informed guesses, the proof of efficacy of the dietary choice would and probably should be informed by genomic approaches. In particular, there is reason to believe that the inflammation associated with CD will itself consume the various nutrients at higher than average levels. These techniques are now sufficiently sensitive to allow testing the effect of the selected dietary strategy following short-term intervention studies. Blood, urine, and feces samples would be required, before and after the study period. Changes in gene expression as monitored by transcriptomic profiles from peripheral blood mononuclear cells show which CD-related genes have modulated activity (89). Urine or fecal samples can be used for metabolomic and proteomic techniques (91, 94). These may be utilized to monitor dietary compliance and also to provide biomarkers relevant to CD progression. The microbiota profile can be estimated from stool samples, and modulation of microbiota will provide important complementary information on whether the dietary selection has the desired effect on slowing disease progression (67, 89, 95). Images from Adobe stockimages and from Ref. (89).

In previous sections, we have identified a number of nutrients that are especially important for CD development and progression. Other nutrients will undoubtedly play an additive role. Systems biology links the interactions among genes, gene products, and environmental factors. As Polytarchou et al. have suggested, there is reason to suggest that this is ready for prime time in CD research (90). It is being increasingly used to refine desirable nutrition for an individual and treat complex human diseases, such as CD. Figure 2 illustrates the way in which these technologies have the potential to revolutionize conventional CD diagnosis and treatment by providing a strong scientific basis for nutritional therapy. As indicated in previous sections and Figure 1, genetic screening will inform the desirability of certain foods or nutrients that will become part of the regular diet (26, 27). The benefit of such a combination of foods may be tested, at an individuals or group level, using transcriptomic approaches (89), while the long-term benefits in protection against disease progression may be interrogated using proteomic and/or metabolomic approaches (91, 92). Changes in the gut microbiota will also be important (93).

Fiocchi has identified four components of IBD pathogenesis as environment, genetics, gut microbiota, and mucosal immune response, which can be defined by the terms “expososome,” “genome,” “microbiome,” and “immunome” (94). None of these act independently, but interact (the “interactome”), resulting in the emergence of IBD. Polytarchou and coworkers (90) have stressed the way in which novel computational methodologies can now integrate high-throughput molecular data. They suggest that a systems biology approach could identify the central regulators in the IBD interactome. This work suggests that identification of key nutrients and their interactions with these central networks, possibly at an individual level, might lead to novel therapeutic approaches to CD.

Metabolomic approaches may provide important tools for understanding the differences between individuals in response to dietary components. For example, Lin and coworkers used a mouse model to investigate the effects of feeding kiwifruit-derived extracts from two different species (96). Not only did they find differences in anti-inflammatory activity between the green (*Actinidia deliciosa*) and gold-fleshed (*Actinidia chinensis*) kiwifruit extracts, but the efficacy of these extracts was modulated by the variant IL-10 genotype. This same mouse model and experimental approach revealed gradual changes in the metabolome as disease developed, particularly decreased levels of very low-density lipoprotein and increased low-density and very low-density lipoproteins and various PUFAs (97). The metabolome also interrelates with the microbiome, suggesting the possibility of utilizing targeted metabolomics for monitoring the consequences of therapeutically manipulating the microbial community as adjunct therapy in CD (98).

Much of the proof of concept of beneficial effects of certain diets in CD patients has been based on animal or tissue culture studies, rather than humans, since there are obvious ethical constraints in working with people. The classic biomarker of inflammation is considered to be CRP, while fecal calprotectin has gained considerable ground. However, as illustrated in the study by Marlow and coworkers, transcriptomics provides a more sensitive and more informative biomarker of beneficial effects of a selected diet in CD than CRP (89).

Research into gastrointestinal cancer has highlighted the importance of biomarkers, especially miRNAs, usually taken from colonic endoscopies. There is considerable interest in the discovery that miRNAs are present in serum in a cell-free state, highlighting the possibility of their potential use as non-invasive biomarkers (99). At this time, extracellular miRNAs have been identified in most biological fluids, including serum, urine, saliva, and breast milk. They are being considered as novel biomarkers, with considerable potential for predicting disease course and response to therapy.

Metabolites reflect the physiological phenotype, providing a molecular readout of the cell status (95). Such measures, taken from various biological fluids, can lead to the identification of “marker metabolites” that differentiate health from disease. In particular, metabonomics (spectroscopy-based metabolic profiling) of fecal extracts has been used to differentiate active from inactive CD. This method comes across as a powerful non-invasive diagnostic tool able to characterize changes in the metabolic profile associated with malabsorption and dysbiosis (100). Specific metabolites that distinguished groups with active from those with inactive disease included N-acetylated compounds and phenylalanine, low-density lipoproteins, and very low-density lipoproteins (101). More generally, metabolomics is being used in human studies to monitor compliance with required dietary changes and also efficacy of the change in slowing disease progression (102).

Proteomics is defined as the large-scale study of proteins, particularly their structures and functions (103). This technique has been used to validate conclusions from other technologies in a series of mouse studies by Barnett and coworkers (20, 46, 104). In studies on green tea polyphenols, proteomic analysis supported the conclusions from microarrays regarding the mechanism of action of the polyphenol-enriched extract (20). Application of the same technology to studies of *n*-3 and *n*-6 PUFAs (arachidonic acid and EPA) showed that the proteomic analysis identified some actions that had not been apparent from transcriptomic studies (46). These authors emphasized the complementary nature of these two approaches and the importance of using the two in concert to better understand the nature of complex diseases such as CD.

The accurate diagnosis of CD has often lagged behind disease presentation. Proteomics is being increasingly used for early and accurate diagnosis of CD as well as for monitoring the course of the disease (92). By providing information on the nature of the disease process, such technology may inform appropriate treatment regimes. Although intestinal tissue will provide the most information, blood, urine, and stool are increasingly providing highly predictive substitutes that are considerably less invasive than the requirement for a colonoscopy.

The toolbox for studying microbiota has become more powerful in recent years, with metagenomics reflecting the study of DNA, metatranscriptomics the total transcribed RNA, and metaproteomics the protein associated with the microbiota. Draft genome sequences have been derived for more than 1,000 human-associated microorganisms derived from the gastrointestinal tract of more than 100 humans (70). Bioinformatics provides an essential tool to tie together these multi-omic analyses of IBD (105).

## Conclusion

There is a great deal written about desirable intakes for various classes of food and nutrients. However, the genetic background of CD would appear to make certain nutrients more important than for the general population, albeit differing somewhat according to the genetics of the individual patient. Such nutrients will be especially important during a flare-up of the disease, but will also be crucial in slowing disease progression. Proof of principle of these assertions has been largely dependent on tissue culture or animal models to date. However, improvements in sensitivity of various technologies, especially transcriptomics, proteomics, and metabolomics, enable non-invasive sampling of humans from blood, urine, and/or stool samples, after relatively short-term human intervention studies. We suggest that there is justification for a paradigm shift in diagnostic tools and nutritional therapy for CD, involving a systems biology approach for implementation (106).

## Conflict of Interest Statement

The author declares that the research was conducted in the absence of any commercial or financial relationships that could be construed as a potential conflict of interest.
